# Fibre tracing in biomedical images: An objective comparison between seven algorithms

**DOI:** 10.1371/journal.pone.0320006

**Published:** 2025-04-10

**Authors:** Youssef Arafat, Cristina Cuesta-Apausa, Esther Castellano, Constantino Carlos Reyes-Aldasoro

**Affiliations:** 1 Department of Computer Science, School of Science and Technology, City St George’s, University of London, London, United Kingdom; 2 Tumour-Stroma Signalling Lab, Universidad de Salamanca, Salamanca, Spain; 3 Integrated Pathology Unit, Division of Molecular Pathology, The Institute of Cancer Research, Sutton, United Kingdom; Bayer Crop Science United States: Bayer CropScience LP,UNITED STATES OF AMERICA

## Abstract

Obtaining the traces and the characteristics of elongated structures is an important task in computer vision pipelines. In biomedical applications, the analysis of traces of vasculature, nerves or fibres of the extracellular matrix can help characterise processes like angiogenesis or the effect of a certain treatment. This paper presents an objective comparison of six existing methodologies (Edge detection, CT Fire, Scale Space, Twombli, U-Net and Graph Based) and one novel approach called *Trace Ridges* to trace biomedical images with fibre-like structures. Trace Ridges is a fully automatic and fast algorithm that combines a series of image-processing algorithms including filtering, watershed transform and edge detection to obtain an accurate delineation of the fibre-like structures in a rapid time. To compare the algorithms, four biomedical data sets with vastly distinctive characteristics were selected. Ground truth was obtained by manual delineation of the fibre-like structures. Three pre-processing filtering options were used as a first step: no filtering, Gaussian low-pass and DnCnn, a deep-learning filtering. Three distance error metrics (total, average and maximum distance from the obtained traces to the ground truth) and processing time were calculated. It was observed that no single algorithm outperformed the others in all metrics. For the total distance error, which was considered the most significative, Trace Ridges ranked first, followed by Graph Based, U-Net, Twombli, Scale Space, CT Fire and Edge Detection. In terms of speed, Trace Ridges ranked second, only slightly slower than Edge Detection. Code is freely available at *github.com/youssefarafat/Trace_Ridges*.

## Introduction

The morphological features of cells and their surrounding environment have been studied with interest in cancer and other conditions [1]. However, it is now recognised that the microenvironment that surrounds these cells has considerable influence [2]. The extracellular matrix (ECM) plays a key role in the development of cancer [3–6] and other diseases [7]. The ECM provides essential structural support [8] to the cellular constituents of tissue. Every organ has a different ECM structure that serves its purpose. The ECM constantly changes its shape and remodels by a process known as ECM proteinases [9,10]. Despite this, the extracellular matrix (ECM) has received less attention [11] than other factors of the microenvironment like macrophages, soluble factors, or vasculature. This could be due to the complicated 3D network of the ECM [12,13], which is formed by many elements such as enzymes, glycoproteins, and collagen.

Whilst visual observation of the extracellular matrix structure can reveal interesting characteristics, it is far too difficult to compare two populations by just observing images. Structural characteristics of the ECM such as the length, distribution, shapes and orientation of the fibre-like structure of these glycoproteins could provide valuable information about the microenvironment or treatments if these were to be objectively analysed. Quantification of the fibres and their characteristics is required and for this purpose, a robust tracing algorithm that identifies individual fibre-like structures is necessary.

There have been many manual, semi-automatic and automatic methodologies proposed to delineate and trace elongated structures some of which are based on algorithms that can be considered more general as they can be applied in a context other than tracing, for instance, skeletonisation [14–16], watershed transform [17,18], medial axis transform [19–21], Scale Space [22,23] and even Edge Detection [24]. However, many of these methodologies have been proposed for tracing in specific applications, which may imply that these have been fine-tuned to the characteristics of the datasets being analysed, e.g., neuron tracing [25–27], retinal images [28–30], angiography [31–33], intravital vasculature observed with confocal, fluorescence or light microscopy [34–36], and extracellular matrix [37,38]. In addition, deep learning architectures like the well-known U-Net [39] have been widely used in image analysis and provided excellent results without the need of hand-crafted features or algorithm. Instead, a combination of pairs of data and labels can be used to train an architecture to perform a specific task, which for the purposes of this paper is the segmentation of fibre-like structures.

In this paper an objective comparison of algorithms that perform tracing of fibre-like structures is presented. Four types of datasets with vastly distinctive characteristics were selected to provide a wide variety of conditions of noise and strength of the fibre structures. The images were hand-delineated to create a ground truth for objective comparisons and to provide training data for the deep learning architecture. Five existing algorithms that have been previously used for similar applications were selected: Edge detection [24], CT Fire[40], Scale Space [22,23], Twombli [38], and Graph Based [41,42]. A U-Net [39] architecture was trained with pairs of patches of data and labels. In addition, a simple, yet quick and effective image processing-based tracing algorithm called *Trace Ridges* is proposed and compared with the other algorithms.

The main contributions of this work are the following:

- The objective comparison of seven fibre tracing methods. Each algorithm was tested in four images with vastly different characteristics. To analyse the effect of noise, three filtering approaches were applied as a pre-processing step for each of the algorithms. Three error metrics and computational processing time were extracted from each of the previous combinations.
- A new tracing pipeline called Trace Ridges was proposed. Trace ridges combines filtering, Watershed transforms, Edge detection and mathematical morphology to trace ridges in an image with fibre-like structures.

## Materials and methods

### Data sets

**Fig 1:**
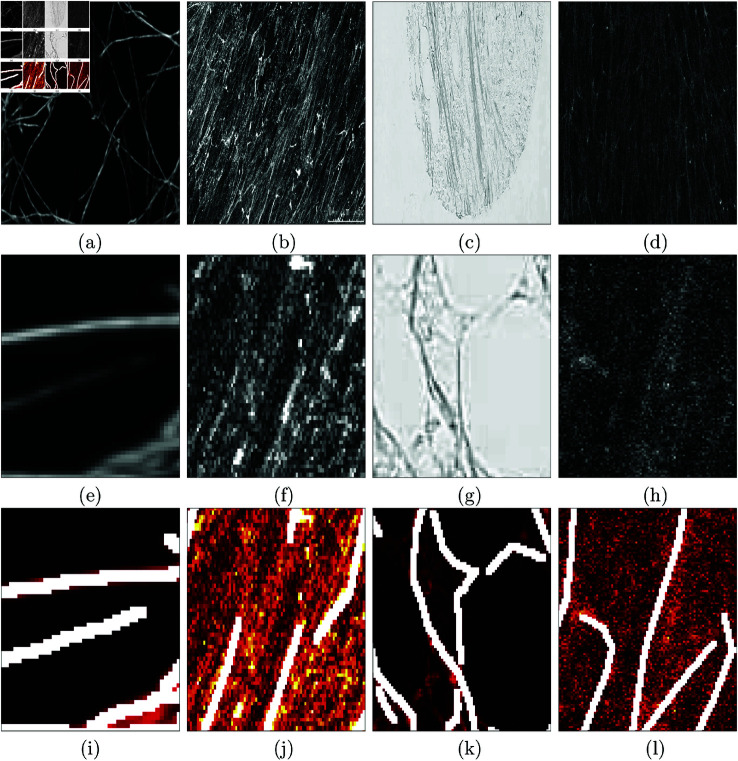
Illustration of the datasets that will be used to compare the tracing algorithms. (a) Second Harmonic Generation Collagen (SHG). (b) Fluorescent Fibronectin (FF). (c) Breast Cancer Biopsy slide (BCB). (d) Disease Mimicking Extra Cellular Matrix (DME). (e-h) Regions of interest (ROI) of the images in (a-d). (i-l) Manually delineated ground truth (GT) for the ROIs of images in (e-h). White lines correspond to the GT and intensities are displayed in a colormap with hot colours (black-red-orange-yellow-white).

Four biological data sets with vastly distinctive characteristics were selected to compare the tracing algorithms (Fig 1). Three of the four data sets were selected as they had been used by the previously published algorithms and were freely available. Details of the data sets have been published previously, but a brief description for each will follow.

- Second Harmonic Generation (SHG) images of tumour bearing mouse mammary glands were published with the CT Fire algorithm [40,43] (Fig 1a). To obtain the images, *in vivo* acquisition was performed through a glass imaging window that was placed at proximity to palpable tumours inside the mammary glands of live 8 weeks old PyMT mice. The excitation wavelength of the SHG images was 890 nm while the emission filter was 445 nm with a 20nm bandwidth. The pulse length was roughly 100 fs. All the SHG images were verified using cellular autofluorescence from the coenzyme Flavin Adenine Dinucleotide, in slides they were verified by white light images of H&E. Images from this data set are clean and fibres are distinguishable from each other.
- Fluorescently labelled fibronectin images (Fig 1b). Fibronectin fibroblasts were cultivated on a gelatine coated surface bonded with glutaraldehyde, used to create the extracellular matrix. Fluorescent images were captured to observe the fibronectin expression. Fibres appear bright and have high intensity, background is darker with lower intensity. Image is available at *github.com/youssefarafat/Trace_Ridges/blob/main/Fluorescent_* *Fibronectin*.
- Breast Cancer Biopsy (BCB) images of collagen (Fig 1c) were published with the Twombli algorithm [38]. The samples were stained with Picrosirius red. The slides were deparaffinised and hydrated prior to the application of Picrosirius red mixture for 60 minutes, after which the samples were rinsed twice in acetic acid followed by alcohol dehydration and mounting. Finally, slides are scanned using a Zeiss Axio Scan.Z1 at 10x magnification. This dataset was chosen as the images are stained with Picrosirius red, including variety to the previous monotone images. Fibres have a filamentous structure, which makes it unique.
- Disease mimicking ECM (DME) (Fig 1d) images of fibroblasts were published with the Graph based algorithm [42]. Normal resting fibroblasts were treated with TGF-B1 cytokine to activate the fibroblast in the tumour micro-environment. The Fibronectin rich matrice was created by exposing recombinant cFn to the Fibronectin null mouse embryo fibroblasts. The cultures finally are decellularised after 7 days. The resultant matrices are viewed with a confocal microscope after they are fluorescently stained, at a 10x/0.45 magnification. Images from this data set are noisy. Brightness variation is low.

### Ground truth

Fibres on (Fig 1a–Fig 1a1d) were manually delineated to create the ground truth to be later used in a quantitative comparison.

### Pre-processing of the data sets

Filtering data sets may reduce the levels of noise and thus improve subsequent steps in a pipeline.

The four data sets present very different noise characteristics, and three different filtering approaches were followed: No pre-processing filtering, low pass filtering with a Gaussian filter and a deep learning de-noising filtering [44]. It should be noted that some methodologies (e.g., Scale Space) include pre-processing steps themselves, but for consistency and ease of comparison all data sets were pre-processed in the same way before applying tracing algorithms.

#### Gaussian filtering.

A Gaussian filter removes noise and detail by combining the values of neighbouring pixels weighted with a Gaussian kernel to replace the original value of a pixel.

A Gaussian filter with a kernel size of 3×3 and a standard deviation value σ= was applied to all the images before applying the tracing algorithms.

#### DnCnn.

DnCnn [44] is a de-noising deep convolution neural network for noisy images. The model adopts residual learning to separate noise from noisy observations. Batch normalisation and residual learning are adopted to improve the de-noising performance. The DnCnn model can handle blind Gaussian de-noising with unknown noise level. The DnCnn model was trained using the images in (Fig 1). Each image was split into half, half was used for training and the other half was unseen. The images were converted to 2-D grayscale where needed as the model only accepts 2-D grayscale images. The model was trained with a learning rate of 0.001 and a batch size of 32.

### Tracing algorithms

The following seven tracing algorithm were compared: Edge Detection (ED) [24], CT Fire (CTF) [40,43], Scale Space (SS) [22,23], Twombli (TW) [38], U-NET [39], Graph Based (GB) [41,42] and the proposed Trace Ridges (TR). They vary in their complexity and methodology with some leveraging filters as part of the pre-processing (GB with Gabor filters, CTF with Curvelet transform for image enhancement). U-Net utilises deep learning techniques. Trace Ridges employs Watershed [17] and Edge Detection for fibre tracing and uses morphological features for image refinement.

#### Edge Detection.

The Canny Edge Detection [24] extracts edges of objects in image by identifying changes in pixel intensities. It employs Gaussian filtering to reduce noise then applies non-maximum suppression to thin the edges. The thresholding process, also known as hysteresis, is performed to exclude or include edges. In this study, the Canny Edge Detection used a filter size of 2×2 to extract edges as proxies for the fibres. Subsequently, the edges were labelled and their metrics extracted.

#### CT Fire.

CT Fire [40,43] was developed to extract and quantify collagen fibres from SHG images. It utilises a curvelet transform (CT) [45] de-noising filter, with Gaussian filtering to remove noise. The curvelet filter represents the image as stacked elements following ridge lines and wavelets, with a frequency wrapping based de-noising technique. Curvelets commonly follow the rule in (Eq 1). The fibre extraction algorithm processes the binary image, with foreground pixels representing fibres. It computes a distance transform to determine the proximity of the foreground pixels to the nearest background pixel, identifies maximal ridges of the smoothed image, and generates nucleation points. After which fibres extend from each nucleation point based on trajectory, followed by removal of short branches.


$$\begin{array}{ccc} \text{width}={\text{length}}^{2} & & \end{array}$$ (1)

#### Scale Space.

Scale Space [22,23] blurs an image across multiple scales to reveal its characteristics. Significant features emerge at lower scales, while finer details are visible at first scales and are progressively smoothed out. Scale Space treats an image as a series of Gaussian smoothed images, represented by the function f(x,y) and Gaussian g(x,y,t), where t denotes the width of the Gaussian filter. First and second derivatives of x and y dimensions are represented as


$$(L_{x},L_{xx},L_{y},L_{yy}),$$

forming a matrix, dictating intensity changes in the image. For Scale Space, the collection of images is represented as L(x,y;t) and Gaussian kernel in (Eq 2). This method was implemented as seen in [36].


$$\begin{array}{ccc} L(x,y;t)=g(x,y;t)\times f(x,y)={\left(\frac{1}{2\pi t}\right)}^{e^{-\frac{x^{2}+y^{2}}{2t}}}\times f(x,y) & & \end{array}$$ (2)

#### Twombli.

Twombli [38] a FIJI macro tool for quantifying fibre-like patterns. It employs existing techniques, including Ridge Detection [46] and AnaMorf [47]. Ridge Detection assesses first and second directional derivatives using Gaussian kernels to detect line points and extract line width with sub-pixel accuracy. Bias is reduced by inverting the Scale Space behaviour of the asymmetric line model. AnaMorf quantifies the structures identified. Twombli also incorporates OrientationJ to obtain the global alignment metric, determining the dominant direction of the fibrial structure. By analysing changes in intensity signal as a function of nearby pixel distances, Twombli constructs segmentation masks using fibre edges.

**U-Net.**

*Properties:*

**Fig 2:**
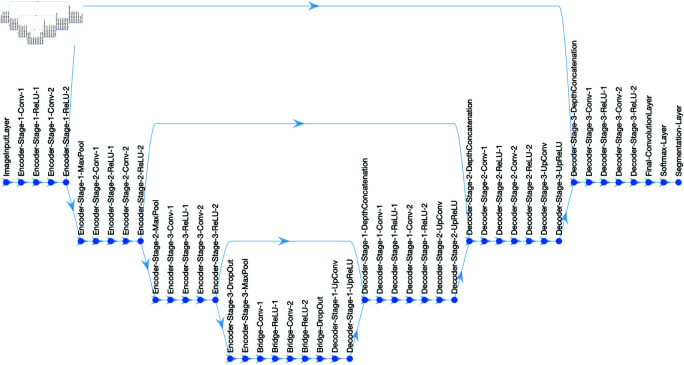
Illustration of the U-Net architecture used to segment the fibres. The architecture consists of three encoder levels and 3 decoder levels.

U-Net [39] is a deep fully convolutional network widely applied in biomedical image segmentation. The architecture compromises of a contracting path for capturing fine details through convolutions, and a symmetric expanding path for accurate localisation. The segmentation map is generated via skip connections between contraction and expanding layers. The network’s architecture is symmetrical to the letter "U". U-Net requires minimal training data and can achieve high performance. The U-Net used in this comparative study (Fig 2) had a depth of 3, consisting of 46 layers, including encoder, bridge, decoder, convolution, softmax and segmentation layers, with skip connections linking the contracting and expanding paths.

**Fig 3:**
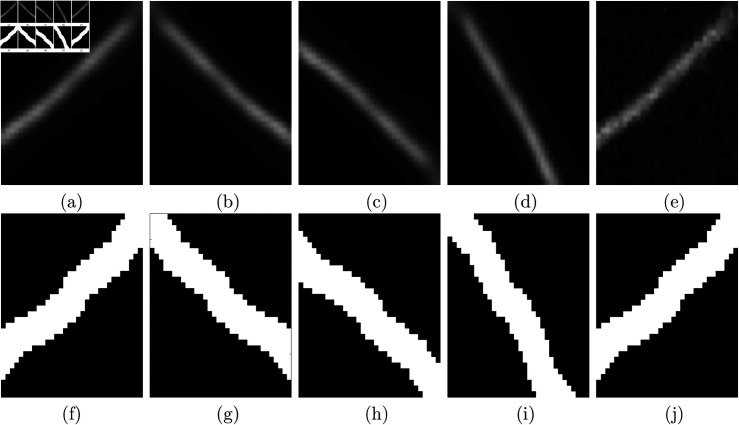
Illustration of the data augmentation approaches used to train the U-Net. (a) Original patch (b) Horizontally flipped (c) Vertically flipped (d) Rotation by 90 degrees (e) Gaussian blurred, with zero mean and a standard deviation of 0.05. (f–j) labels corresponding to augmented patches. It should be noted that (j) is the same as (f) as the Gaussian blur does not affect the label.

*U-Net Training*: Initially, various training parameters were tested, including batch size (16 and 32), and learning rate (0.0001 and 0.001), to optimise training parameters and select the best-performing network. Each image from (Fig 1a–Fig 1d) was split into halves for training and testing. BCB Collagen image (Fig 1c) was complemented to highlight fibre features in white, and background in black. To increase the number of images for training, augmentation was employed. Augmentation techniques (Fig 3) included horizontal and vertical flipping, rotation by 90 degrees and addition of Gaussian noise. A total of 8910 images of size 32x32 were used for training. The U-Net was trained with a batch size of 16, learning rate of 0.0001 and the "adam" optimisation algorithm, for 15 epochs. Training duration was 15 minutes.

#### Graph based.

The Graph-based algorithm [41,42] combines fast discrete curvelet transform filters to detect curvilinear anisotropic objects, in combination with a fibre extraction algorithm. The algorithm uses Gabor filters to enhance fibres that have been observed with confocal microscopy. The algorithm offers flexibility by detecting various fibre elements at different frequencies and orientations, avoiding translation and rotation errors. The algorithm associates graph networks with fibre morphological skeletons, followed by fibre pruning and post-processing fibre re-connection. Two graph types are used to measure fibre properties: one representing fibre crosslinks or ends, and the other simplifying the first graph into a skeleton. These representations were used to extract geometrical and topological properties from the confocal images. Then, specific properties, e.g., orientation for Gabor and fibre length as graph parameters were used to relate to the physical properties of the fibres.

#### Trace Ridges.

**Fig 4:**
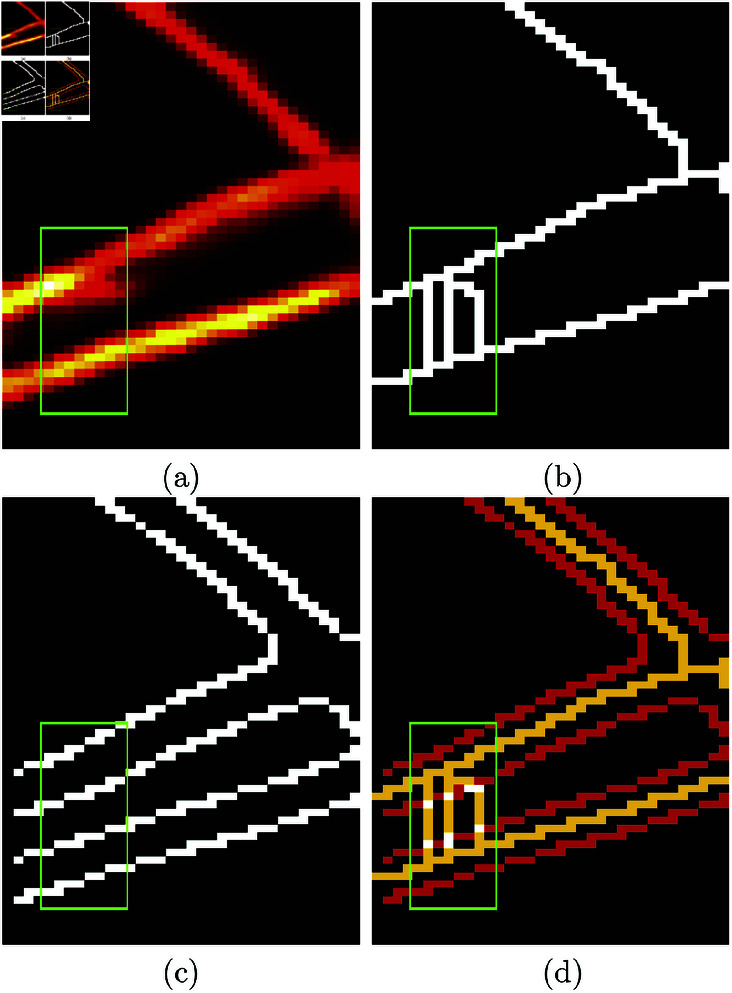
Illustration of the Trace Ridges methodology. (a) Region of interest of one SHG image that shows some strong ridges and a region with some noise highlighted with a green rectangle. (b) Output of a Watershed transform, which traces the strong ridges but also some smaller ones that are not of interest. (c) Edges detected with Canny’s algorithm highlight the sides of the strong ridges. (d) The combination of Edge Detection (red lines) with Watershed (yellow lines). White pixels correspond to the overlap of both techniques. The watershed will be broken by removing these overlapping pixels.

In this paper, a methodology called *Trace Ridges* is proposed. The methodology combines Watershed transform and Edge detection. First, the Watershed is used to delineate ridges. The *main* ridges will correspond to the fibres to be traced, but in addition, *minor* ridges, product of the well-known over segmentation of watersheds [48,49], will appear as coming down from the main ridges. Then, the Edge detection process detects regions where there are sudden changes of intensity, i.e., close to the ridges, ideally one on each side of the centre line of the ridge. The intersection between the edges and watersheds is used to *break* those *minor* ridges that run down from a main ridge through the sides of the basins, thus separating them from the major ridges. In this way, the well-known problem of over-segmentation of Watershed is counteracted and the methodology prioritises longer ridges along the brighter sections of an image for cleaner results. The combination of these two steps of Trace Ridges is illustrated in (Fig 4a). The region contains three strong and bright ridges with a small noisier region highlighted with a green rectangle. The results of Watershed and Edge Detection are shown separately in (Fig 4b, 4c) and the overlap in (Fig 4d) where the edges are shown in red, and the watershed is shown in yellow. The points where the two overlap are shown as white. These points are discarded thus breaking the small watersheds and maintaining the main ridges. Next, the edges and small segments with a major axis length smaller than a threshold (5 pixels) are removed. Segments that lie within darker regions are also removed as it may be possible that there are watersheds in dark regions that are hardly visible and not of interest.

In some cases, a thicker ridge may produce two parallel watersheds with a very small hole in between. These cases are identified by their Euler characteristic [50]. Only very small holes are filled in, and then the resulting object is thinned, this process leaves a trace that runs through the position of that small hole.

Also, there are cases where Watershed will not detect a ridge, specifically when both sides of the ridge feed to a single basin surrounding it. Those cases are detected by using only Edge detection, filling in the space between the edges, and then thinning it to create a single line where the ridge is located. In some cases, Edge Detection results in only one edge for a ridge, these are discarded and treated as low level ridges or noise.

Finally, Trace Ridges discards small and curved fibres and keeps longer and straighter lines. In the case that Trace Ridges detects less than 100 fibres from the missed ridges. Trace Ridges retains from those ridges only those that have a major axis length above 15 pixels and a minor axis length less than 10 pixels. If the number of new fibres detected is higher, a stricter condition is applied whereby the fibres must have a major axis length of at least 25 pixels and the minor axis length must be less than 10 pixels.

Once all the fibres are detected, Trace Ridges combines the ridges detected with the combination of Watershed and Edge Detection as well as the missed ridges detected by Edge Detection, prioritising longer and brighter fibres.

Many morphological metrics can be extracted from the traced lines for instance, orientation, length, width, count, eccentricity, and mean intensities. Furthermore, the gaps between fibres can be calculated and from there, metrics such as gap area and largest gaps can be extracted.

Finally, three dimensional datasets are projected to two dimensions with a maximum intensity projection.

### Quantitative comparison with distance metrics

**Fig 5:**
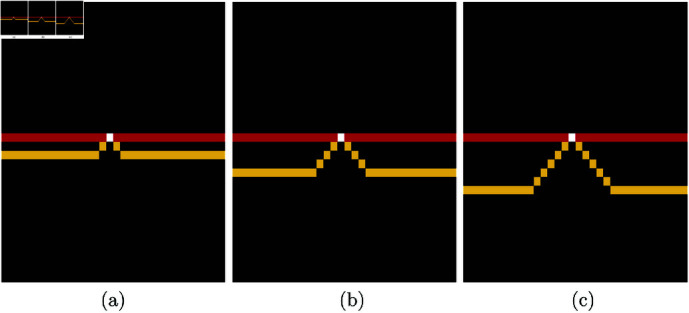
Illustration of three cases of two lines where the pixel-wise metrics of true positives, true negatives, false positives, false negatives are identical, yet the distance between the lines is different.

Quantitative comparisons between the GT and results of the algorithms were calculated using distance metrics as has been previously reported [36]. It is important to highlight that traditional pixel-wise metrics such as accuracy, true/false positive rate, sensitivity, and specificity are not suitable for comparing traces against a ground truth. This is illustrated in (Fig 5), which shows three cases of lines that overlap in one single pixel and then extend from there in very different conditions. Whilst the cases are quite different, pixel-wise metrics would identify the same number of true positives, one, in all cases. Similarly, the true negatives (black pixels) could be similar and not useful to distinguish between cases. Pixel-wise metrics are useful with larger structures such as nuclei and cells where the comparison is between areas and not how far from each other the lines can be.

**Fig 6:**
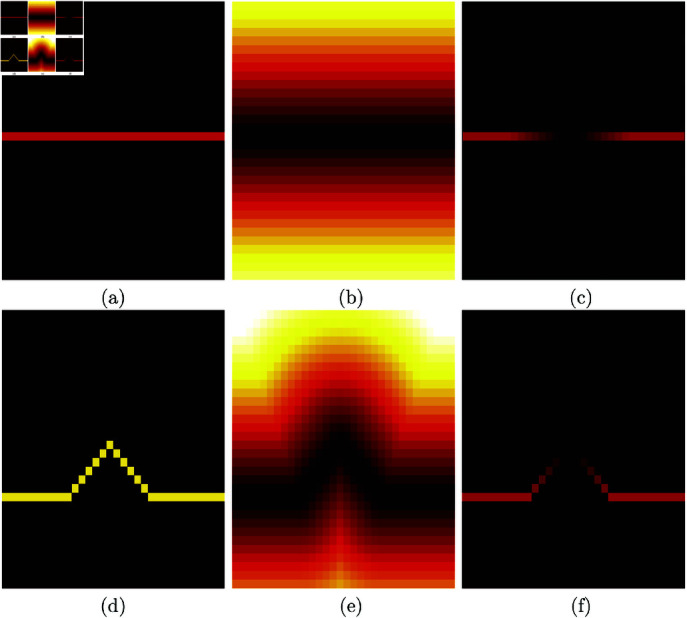
Illustration of the distance error measurement between the two lines of (Fig 5c). (a, d) Two lines that overlap only at a single point. (b) Distance map computed from the red line in (a). Distances follow a hot colour map: black-red-orange-yellow-white. (c) Product of the distance map shown in (e) with the line shown in (a). (e) Distance map computed from the yellow line in (d). (f) Product of the distance map shown in (b) with the yellow line shown in (d).

**Fig 7:**
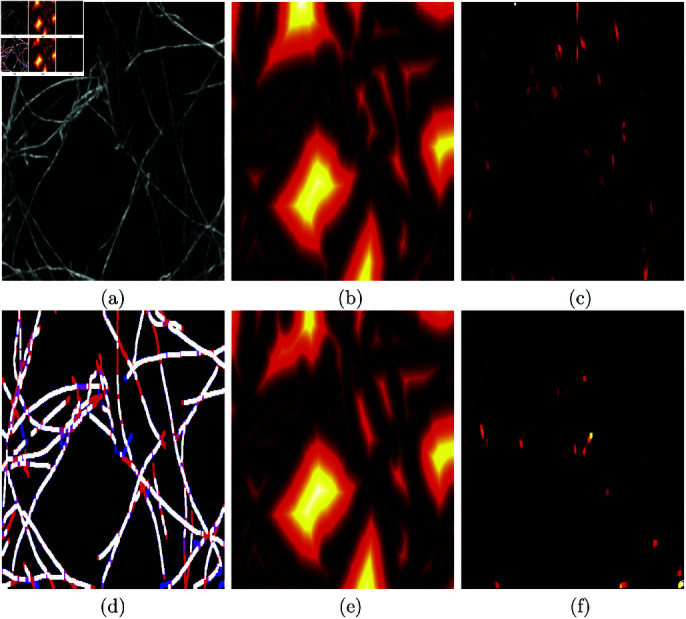
Illustration of the calculation of the error distance maps on the Second Harmonic Generation image shown in (Fig 1a). (a) One representative image with a variety of fibres. (b) Distance map calculated from the traces calculated with Trace Ridges. (c) Product of the ground truth and the distance map of (b). (d) Comparison between the results of Trace Ridges and the ground truth. White lines indicate pixels from GT and trace. Blue lines correspond to Trace Ridges. Red lines are from the GT. (e) Distance map calculated from the ground truth. (f) Product of the traces from Trace Ridges and the distance map of (e). It should be noticed how the cases where the lines overlap (white) in (d) do not appear in any of the distance maps (c, f) whilst the lines that do not overlap (blue and red) appear in the distance maps with increased brightness as they are further away from the lines generated by the opposite method.

Calculation of the distance metrics has been previously described [36] but will be briefly here for completeness and illustrated with a simple example in (Fig 6) and the Second Harmonic Generation image in (Fig 7). First, a distance map from each of the set of traces to be compared was calculated (Fig 6b, Fig 6e) and (Fig 7b, 7e). Then the map of one set was multiplied by the opposite set. Thus, the traces that are further away from the traces from the opposite set will be in regions of higher distance and close to their opposites in regions of low distance (Fig 6c, Fig 6f) and (Fig 7c, Fig 7f).

From those two maps many metrics can be calculated, for instance, the average distance error per trace, maximum distance error per trace and total distance error.

**Table 1:** Quantitative Comparison by total error distance in pixels of the following Tracing methods: Edge Detection (ED), CT Fire (CTF), Scale Space (SS), Twombli (TW), U-Net, Graph based (GB) and Trace Ridges (TR), with three filtering options on the following images: Disease Mimicking ECM (DME), Second Harmonic (SHG), Fluorescent Fibronectin (FF) and Breast Cancer Biopsy (BCB) images. In addition to the errors per image, average per filtering option, average of the methodology and rank are presented. Results are sorted by rank with the worst results at the top (7) and best results at the bottom (1).

Method		Image							
	**Filter**	**DME**	**SHG**	**FF**	**BCB**	**Average**	**Rank**	**avg/Method**	**Rank**
ED	No Filt	947,550	35,905	754,120	224,120	490,424	21	429,173	7
	Gaus Filt	834,660	36,248	714,530	208,090	448,382	20		
	DnCnn	620,970	32,591	612,600	128,690	348,713	19		
CTF	No Filt	311,580	13,594	232,140	83,653	160,242	16	171,529	6
	Gaus Filt	342,290	22,687	269,290	79,694	178,490	18		
	DnCnn	330,190	29,437	266,950	76,848	175,856	17		
SS	No Filt	206,620	15,654	318,770	72,760	153,451	14	146,592	5
	Gaus Filt	209,020	15,493	326,510	75,720	156,686	15		
	DnCnn	215,930	15,334	212,400	74,890	129,639	7		
TW	No Filt	218,870	14,160	286,120	60,969	145,030	11	141,988	4
	Gaus Filt	186,810	14,328	280,020	49,700	132,715	8		
	DnCnn	248,420	14,087	280,280	50,091	148,220	12		
UNET	No Filt	180,610	12,938	129,040	47,940	92,632	1	129,457	3
	Gaus Filt	268,560	12,119	248,620	45,840	143,785	9		
	DnCnn	292,440	12,220	260,070	43,090	151,955	13		
GB	No Filt	247,040	14,953	209,990	103,750	143,933	10	123,648	2
	Gaus Filt	191,540	14,693	203,640	68,019	119,473	6		
	DnCnn	154,756	19,502	188,520	67,372	107,538	2		
TR	No Filt	208,580	9,141	168,170	70,320	114,053	5	112,120	1
	Gaus Filt	198,810	9,537	166,690	63,520	109,639	3		
	DnCnn	195,270	13,727	162,760	78,910	112,667	4		

**Table 2:** Quantitative Comparison by average error distance in pixels of the following Tracing algorithms; Edge Detection (ED), CT Fire (CTF), Scale Space (SS), Twombli (TW), U-Net, Graph based (GB) and Trace Ridges (TR) with three filtering options on Disease Mimicking ECM (DME), Second Harmonic (SHG), Fluorescent Fibronectin (FF) and Breast Cancer Biopsy (BCB) images. In addition to errors per image, average per filtering option, average of the methodology and rank are presented. Results are sorted by rank with the worst results at the top (7) and best results at the bottom (1).

Method		Image							
	**Filter**	**DME**	**SHG**	**FF**	**BCB**	**Average**	**Rank**	**avg/Method**	**Rank**
GB	No Filt	19.3	15.0	17.7	10.5	15.6	21	12.0	7
	Gaus Filt	15.3	4.1	17.3	6.6	10.8	18		
	DnCnn	11.0	7.4	13.0	7.0	9.6	13		
UNET	No Filt	10.7	12.9	10.7	8.2	10.6	16	11.0	6
	Gaus Filt	11.8	13.6	14.4	7.5	11.8	20		
	DnCnn	12.7	9.7	14.3	6.1	10.7	17		
CTF	No Filt	8.6	15.7	14.3	8.4	11.7	19	9.6	5
	Gaus Filt	11.4	3.8	11.4	7.9	8.6	11		
	DnCnn	9.5	6.4	10.1	7.5	8.4	9		
SS	No Filt	10.7	5.2	16.1	8.6	10.1	15	9.5	4
	Gaus Filt	10.4	4.7	15.8	8.2	9.8	14		
	DnCnn	10.7	5.0	10.4	8.2	8.6	10		
TW	No Filt	10.0	5.0	10.6	6.9	8.1	8	8.2	3
	Gaus Filt	9.7	4.7	10.3	5.7	7.6	5		
	DnCnn	14.5	5.5	10.3	5.4	8.9	12		
TR	No Filt	11.5	3.1	9.8	7.6	8.0	6	7.8	2
	Gaus Filt	10.2	3.4	8.7	6.7	7.3	3		
	DnCnn	10.5	4.3	9.3	8.3	8.1	7		
ED	No Filt	8.4	4.8	9.4	6.4	7.3	2	7.2	1
	Gaus Filt	8.5	4.9	9.1	6.6	7.3	4		
	DnCnn	8.3	5.1	8.8	5.7	7.0	1		

## Results and discussion

In this work, images of fibre-like structures were traced by seven different image processing algorithms and were filtered by three different methods. To compare and observe which method traces fibres better, four quantitative measurements were calculated: the total distance error, average distance error and maximum distance error, as well as computational time. The distance errors are measured by comparing how close the trace results of each algorithm were compared to the manually delineated ground truth.

### Quantitative comparison of the algorithms

#### Total distance error.

The first analysis focuses on total distance errors, the accumulation of the distance errors from GT to algorithm trace generated by the distance maps, as explained. Qualitative results are summarised in Table 1, comparing algorithms with and without filtering. Filtering notably improves Edge Detection, Scale Space, Twombli, Graph Based, and Trace Ridges. DnCnn enhances Edge Detection, Scale Space, and Graph Based, while Gaussian filtering benefits Twombli and Trace Ridges. However, filtering worsens Scale Space with Gaussian and Twombli with DnCnn. U-Net and CT Fire exhibit increased errors post-filtering, likely due to U-Net training on non-filtered images and CT Fire’s inherent filtering. Notably, U-Net’s performance improved when DnCnn was used on the BCB and SHG images, while U-Net with DnCnn yielded larger errors for the other images, impacting its overall performance. Considering filtering + algorithm pairs as separate methods, U-Net without filtering performs best, followed by Graph Based with DnCnn, Trace Ridges with Gaussian filtering, Trace Ridges with DnCnn and Trace Ridges with no filtering. However, averaging total distance errors across all images regardless of filtering reveals Trace Ridges as the top-performing method, followed by Graph Based, U-Net, Twombli, Scale Space, CT Fire and Edge Detection.

#### Average distance error.

This quantitative metric calculates total errors divided by the number of traces to provide an average, as shown in Table 2. Filters enhance the performance of various methods including Edge Detection, CT Fire, Scale Space, Twombli, Graph based and Trace Ridges. However, filters do not improve U-Net’s values due to its training on non-filtered images. Notably DnCnn improves U-Net’s performance for the DME and BCB images, while Gaussian filtering benefits only the BCB image. Filtering yields consistent results for Edge Detection, with DnCnn slightly improving it. Both Gaussian and DnCnn filters significantly enhance CT Fire. Gaussian filtering marginally improves Scale Space, while DnCnn enhances it. Twombli’s results are improved by Gaussian filtering but worsened by DnCnn, in agreement with the total distance error metric. Filtering improves the Graph based method, with DnCnn yielding the best performance. Gaussian filtering improves Trace Ridges, but DnCnn slightly increases the value, impacting performance. Filtering + algorithm pairs are ranked, with Edge Detection + DnCnn performing best, followed by Edge Detection + no filter, Trace Ridges + Gaussian, Edge Detection + Gaussian and Twombli + Gaussian. Analysing averages per method, Edge Detection ranks highest, followed closely by Trace Ridges and Twombli. Edge Detection performs well due to the proximity of its traces to the GT, although it detects unwanted edges as fibres. Conversely, Trace Ridges, while achieving the best total distance error and the second-best average, is considered a more robust and accurate tracing method.

#### Maximum distance error.

This metric examines the maximum singular error in each image, as shown in Table 3. Filtering enhances the performance of Edge Detection, CT Fire, Scale Space, U-Net, Graph Based and Trace Ridges. Filtering hinders Twombli. Gaussian filtering produces comparable results to no filtering for Edge Detection, while DnCnn enhances results. CT Fire shows slight improvement with Gaussian and more significant improvement with DnCnn, both Gaussian and DnCnn improve Scale Space, with DnCnn yielding the lowest maximum error. Interestingly, Gaussian filtering improves U-Net but worsens with DnCnn filtering, DnCnn hinders the performance of U-Net on the SHG image, heavily impacting the average. For Graph based, DnCnn’s impact resembles that of no filtering, whereas Gaussian improves results, both Gaussian and DnCnn improve Trace Ridges, with Gaussian having a similar effect to DnCnn but slightly lesser. Among filtering + algorithm pairs, Graph based + Gaussian performs the best, followed by U-Net + Gaussian, U-Net + no filtering, Edge Detection + DnCnn and U-Net + DnCnn. Observing averages per method, U-Net achieves the best results, followed by Graph Based and Edge Detection. However, Trace Ridges does not perform as well in this metric due to its preference for brighter and longer fibres, resulting in higher singular maximum errors. While this metric provides insights, total and average distance errors are better indicators of algorithm robustness and accuracy.

**Table 3:** Quantitative Comparison by maximum error distance in pixels of the following Tracing algorithms; Edge Detection (ED), CT Fire (CTF), Scale Space (SS), Twombli (TW), U-Net, Graph based (GB) and Trace Ridges (TR) with three filtering options on Disease Mimicking ECM (DME), Second Harmonic (SHG), Fluorescent Fibronectin (FF) and Breast Cancer Biopsy (BCB) images. In addition to errors per image, average per filtering option, average of the methodology and rank are presented. Results are sorted by rank with the worst results at the top (7) and the best results at the bottom (1).

Method		Image							
	**Filter**	**DME**	**SHG**	**FF**	**BCB**	**Average**	**Rank**	**avg/Method**	**Rank**
SS	No Filt	65.2	50.4	69.6	202.4	96.9	21	94.9	7
	Gaus Filt	63.6	55.6	66.4	191.8	94.4	20		
	DnCnn	61.4	57.4	59.2	195.5	93.4	19		
CTF	No Filt	68.9	89.0	81.0	73.6	78.1	16	77.5	6
	Gaus Filt	57.5	98.5	75.5	82.7	78.5	17		
	DnCnn	58.4	96.5	75.7	73.1	75.9	15		
TR	No Filt	68.5	55.0	95.2	102.1	80.2	18	76.1	5
	Gaus Filt	84.3	50.4	91.9	70.4	74.3	14		
	DnCnn	76.3	48.8	88.2	81.7	73.7	13		
TW	No Filt	62.4	65.8	57.8	68.7	63.7	8	66.3	4
	Gaus Filt	69.8	66.9	60.5	60.5	64.4	9		
	DnCnn	79.2	89.0	62.7	51.8	70.7	12		
ED	No Filt	45.4	59.4	52.3	119.7	69.2	10	66.3	3
	Gaus Filt	46.1	59.4	52.3	119.7	69.4	11		
	DnCnn	50.3	86.0	58.3	46.1	60.2	4		
GB	No Filt	58.9	72.1	65.8	56.2	63.2	7	60.7	2
	Gaus Filt	59.6	53.8	62.9	47.8	56.0	1		
	DnCnn	71.6	55.8	70.6	53.2	62.8	6		
UNET	No Filt	58.8	63.4	66.4	50.9	59.9	3	59.8	1
	Gaus Filt	56.1	63.4	57.6	50.4	56.9	2		
	DnCnn	57.2	89.0	56.0	47.8	62.5	5		

#### Time.

The processing time for each trace of every algorithm with various filter combinations on each image was recorded and is presented in Table 4. Surprisingly, filtering improved the speed of every algorithm, with DnCnn consistently achieving the fastest results across all algorithms and filters. However, Edge Detection exhibited consistent speed with Gaussian and no filtering, and Gaussian filtering slowed down Scale Space. Each subsequent algorithm and filter pair in Table 4 generally performed better and faster, except for Graph Based with DnCnn, which outperformed Twombli. U-Net with DnCnn was faster than Trace Ridges with Gaussian or no filtering. Edge Detection was the fastest algorithm overall, followed closely by Trace Ridges, which is known for its efficiency. U-Net was ×3 slower than Trace Ridges, followed by Scale Space and CT Fire. Speed variations were significantly and heavily influenced by image noise levels, with noisier images (FF and DME) taking longer to process than less noisy images.

**Table 4:** Quantitative Comparison by time taken in seconds of the following Tracing algorithms; Edge Detection (ED), CT Fire (CTF), Scale Space (SS), Twombli (TW), U-Net, Graph based (GB) and Trace Ridges (TR) with three filtering options on Disease Mimicking ECM (DME), Second Harmonic (SHG), Fluorescent Fibronectin (FF) and Breast Cancer Biopsy (BCB) images. In addition to time taken per image, average per filtering option, average of the methodology and rank are presented. Results are sorted by rank with the worst results at the top (7) and the best results at the bottom (1).

Method		Image							
	**Filter**	**DME**	**SHG**	**FF**	**BCB**	**Average**	**Rank**	**avg/Method**	**Rank**
GB	No Filt	1387.0	88.0	550.0	146.0	542.75	21	433.08	7
	Gaus Filt	836.0	55.0	664.0	153.0	427.00	20		
	DnCnn	527.0	84.0	573.0	134.0	329.50	16		
TW	No Filt	383.0	211.0	777.0	306.0	419.25	19	385.92	6
	Gaus Filt	681.0	340.0	438.0	152.0	402.75	18		
	DnCnn	520.0	303.0	380.0	140.0	335.75	17		
CTF	No Filt	180.0	60.0	145.0	77.0	115.50	15	102.42	5
	Gaus Filt	167.0	57.0	110.0	71.0	101.25	14		
	DnCnn	135.0	49.0	113.0	65.0	90.50	13		
SS	No Filt	98.6	4.8	72.7	45.4	55.40	11	55.78	4
	Gaus Filt	86.0	5.2	71.8	82.5	61.36	12		
	DnCnn	87.0	5.1	68.0	42.2	50.57	10		
UNET	No Filt	23.0	5.2	21.1	20.1	17.33	9	12.23	3
	Gaus Filt	20.1	5.0	18.9	23.7	16.92	8		
	DnCnn	2.5	2.2	2.4	2.6	2.44	5		
TR	No Filt	8.0	0.5	4.0	2.7	3.81	7	3.00	2
	Gaus Filt	4.5	0.8	3.1	3.0	2.87	6		
	DnCnn	4.0	0.6	2.9	1.8	2.32	4		
ED	No Filt	1.2	0.1	0.5	0.5	0.58	2	0.53	1
	Gaus Filt	0.7	0.5	0.5	0.6	0.58	3		
	DnCnn	0.8	0.1	0.4	0.4	0.43	1		

### Visual assessment of the algorithms

**Fig 8:**
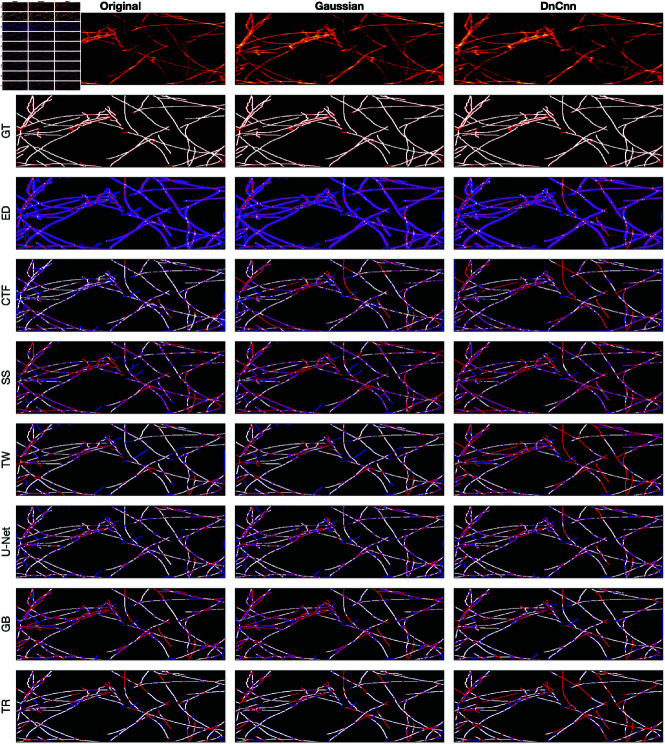
Comparison of tracing algorithms on SHG image (full view) [40]. Image was applied to algorithms with three different filters: Original/no filtering, Gaussian and DnCnn. Data corresponds to the tracing images; GT is the manually delineated ground truth. For the evaluation of Tracing Algorithms, the GT fibres are represented in red, algorithm tracing is depicted in blue and areas where they overlap is white.

**Fig 9:**
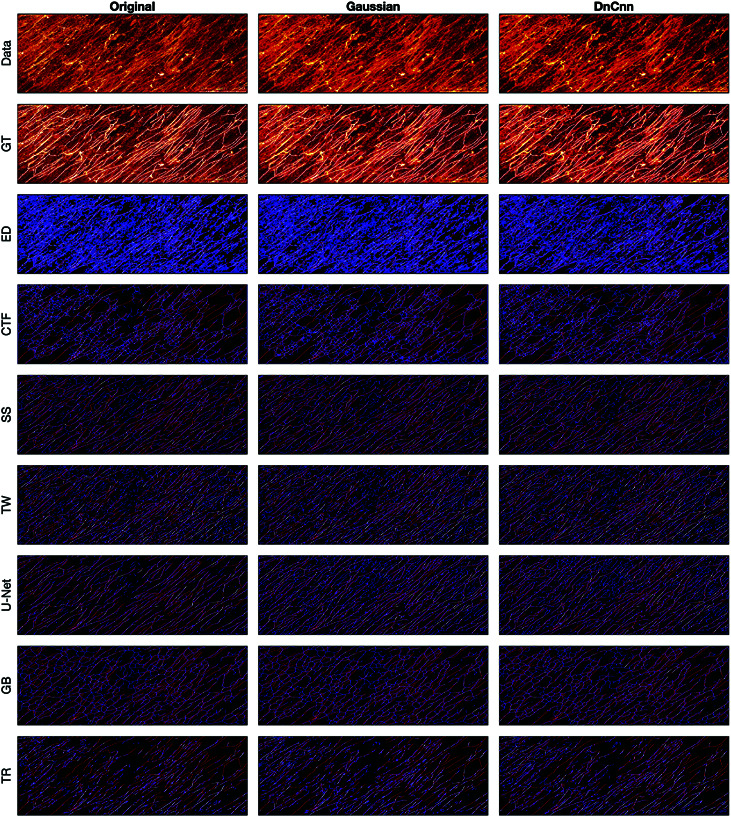
Comparison of tracing algorithms on FF image (full view). Image was applied to algorithms with three different filters: Original/no filtering, Gaussian and DnCnn. Data corresponds to the images traced; GT is the manually delineated ground truth. For the evaluation of Tracing Algorithms, the GT fibres are represented in red, algorithm tracing is depicted in blue and areas where they overlap is white.

**Fig 10:**
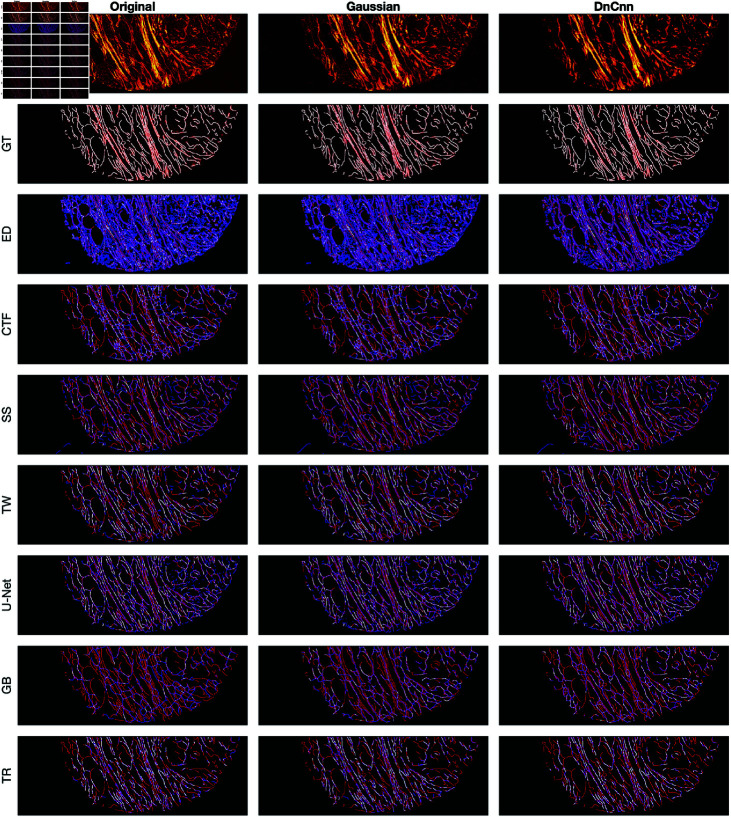
Comparison of tracing algorithms on Picrosirius red Breast Cancer Biopsy image (full view) [38]. Image was applied to algorithms with three different filters: Original/no filtering, Gaussian and DnCnn. Data corresponds to the images traced; GT is the manually delineated ground truth. For the evaluation of Tracing Algorithms, the GT fibres are represented in red, algorithm tracing is depicted in blue and areas where they overlap is white.

**Fig 11:**
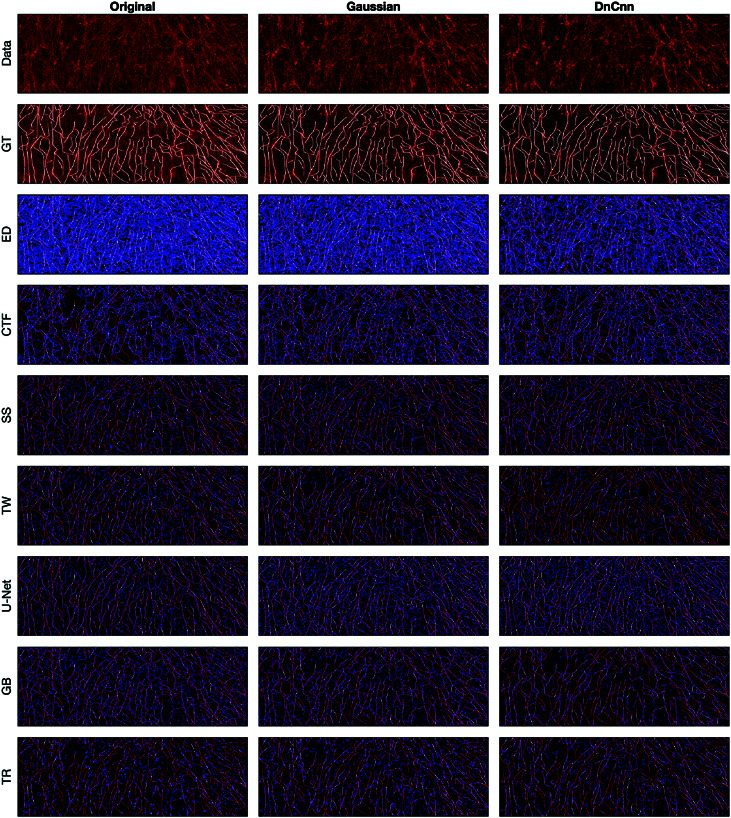
Comparison of tracing algorithms on DME image (full view) [42]. Image was applied to algorithms with three different filters: Original/no filtering, Gaussian and DnCnn. Data corresponds to images traced; GT is the manually delineated ground truth. For the evaluation of Tracing Algorithms, the GT fibres are represented in red, algorithm tracing is depicted in blue and areas where they overlap is white.

**Fig 12:**
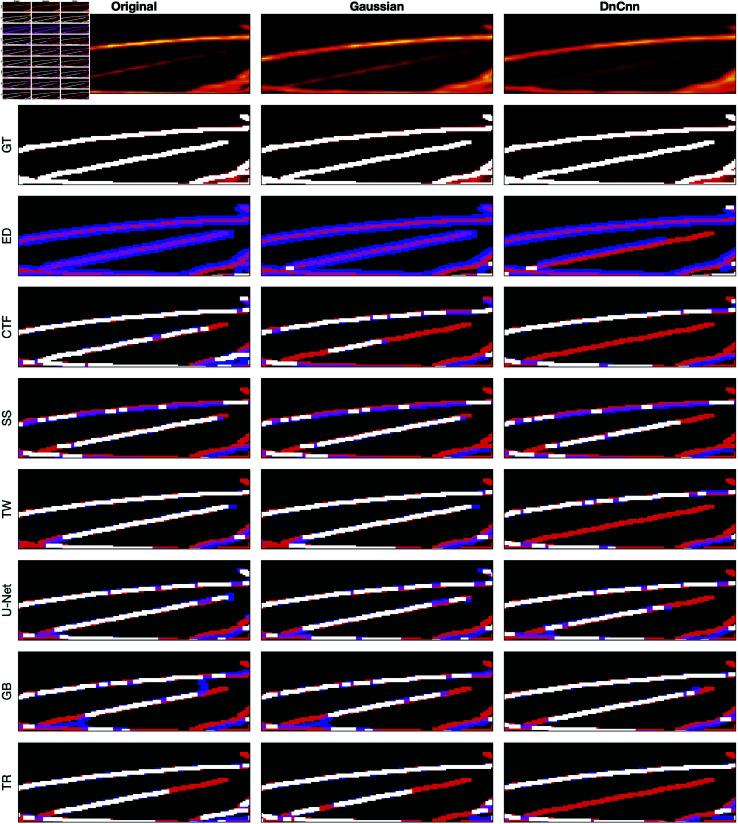
Comparison of tracing algorithms on SHG image (region of interest) [40]. Image was applied to algorithms with three different filters: Original/no filtering, Gaussian and DnCnn. Data corresponds to the images traced; GT is the manually delineated ground truth. For the evaluation of Tracing Algorithms, the GT fibres are represented in red, algorithm tracing is depicted in blue and areas where they overlap is white.

**Fig 13:**
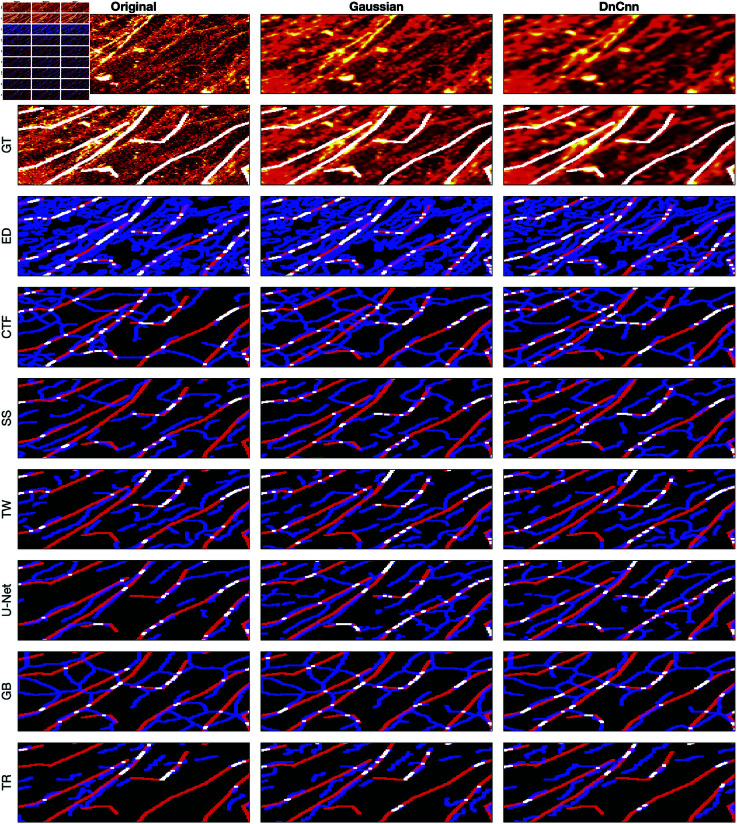
Comparison of tracing algorithms on FF image (region of interest). Image was applied to algorithms with three different filters: Original/no filtering, Gaussian and DnCnn. Data corresponds to images traced; GT is the manually delineated ground truth. For the evaluation of Tracing Algorithms, the GT fibres are represented in red, algorithm tracing is depicted in blue and areas where they overlap is white.

**Fig 14:**
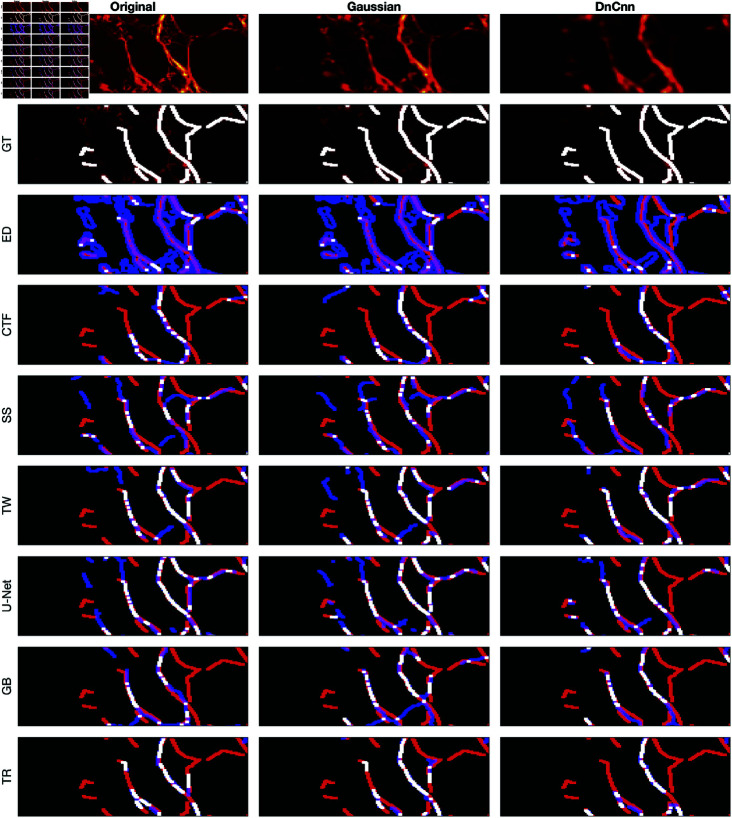
Comparison of tracing algorithms on Picrosirius red Breast Cancer Biopsy image (region of interest) [38]. Image was applied to algorithms with three different filters: Original/no filtering, Gaussian and DnCnn. Data corresponds to images traced; GT is the manually delineated ground truth. For the evaluation of Tracing Algorithms, the GT fibres are represented in red, algorithm tracing is depicted in blue and areas where they overlap is white.

**Fig 15:**
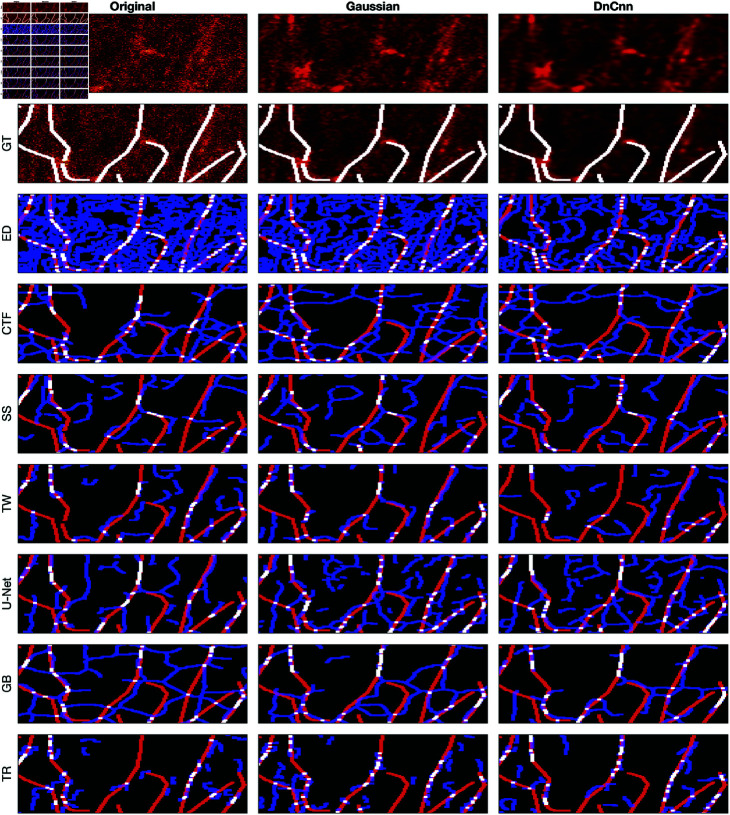
Comparison of tracing algorithms on DME image (region of interest) [42]. Image was applied to algorithms with three different filters: Original/no filtering, Gaussian and DnCnn. Data corresponds to images traced; GT is the manually delineated ground truth. For the evaluation of Tracing Algorithms, the GT fibres are represented in red, algorithm tracing is depicted in blue and areas where they overlap is white.

A comparison between the results and the GT is presented in (Fig 8), (Fig 9), (Fig 10), (Fig 11), (Fig 12), (Fig 13), (Fig 14) and (Fig 15) with the GT in red, the results in blue. White lines correspond to pixels where the result of the algorithm and GT overlap, i.e., correct traces. It should be noted that in some cases a purple colour may be perceived, but this is an artefact when a blue line is remarkably close to a red line.

#### Second harmonic generation.

The SHG is a clear image, detected and missed fibres are easily identifiable. Edge Detection (Fig 8) consistently produces duplicate lines for each fibre. From (Fig 12), DnCnn application improves the tracing, increasing white regions compared to the original. CT Fire algorithm has demonstrated notable success on the original image (Fig 8) but struggles with filtered images, evidenced by decreased number of white lines regions (Fig 12), suggesting hindrance by filtering. Scale Space remains unaffected by filtering, showing consistent results (Fig 12). Scale Space does not perform well. Twombli exhibits sporadic traces and misses major fibres (Fig 8). Application of DnCnn appears to worsen Twombli’s performance (Fig 12). U-Net performs well, with significant white line regions (Fig 8). Filtering does not visibly affect U-Net’s segmentation. The Graph based method misses several fibres shown in red. It also detects spurious objects with DnCnn application (Fig 8). Trace Ridges shows the best visual performance, with the highest number of correct fibre regions (Fig 8). However, visually, Trace Ridges with DnCnn performs the worst amongst filter methods (Fig 12).

#### Fluorescent fibronectin.

For the Fluorescent Fibronectin image (Fig 9), several notable findings emerge. Despite the increased noise on the image as compared to the previous example (SHG), similar trends persist, that is, both CT Fire and Edge Detection generate a large number of traces, more than there are actual fibres. Filtering enhances Edge Detection (Fig 13), but degrades the results CT Fire. Scale Space and Trace Ridges yield fewer traces, with Scale Space detecting darker fibres and Trace Ridges favouring the bright ones. Trace Ridges exhibits fewer spurious lines (blue) and more white regions, indicative of its strict nature (Fig 13), showing improvement with image filtering. Graph based method and Twombli exhibit over-segmentation, visible as blue regions (Fig 9) and (Fig 13). Pre-filtering notably improves their performance. Conversely, U-Net’s results with filtered images are inaccurate, as it was trained on non-filtered images, yielding optimal results with the original image as is visible in (Figs 9 and 13).

#### Breast cancer biopsy.

For the BCB image, similar trends persist, with Edge Detection over-segmenting and producing inaccuracies, as seen in (Fig 10) and (Fig 14). However, pre-filtering results in cleaner images and reduces inaccuracies. CT Fire misses major fibres while detecting noise as spurious objects, evident in (Fig 10) and (Fig 14). Filtering enhances the algorithm’s performance, as observed in (Fig 14). Scale Space shows inaccuracies, detecting noise and spurious objects, contrary to its usual strict behaviour, as shown in (Fig 14). Filter application does not alter the results. Twombli shows notable performance, especially in detecting fibres at the top right corner in (Fig 14). Gaussian filtering improves Twombli, while DnCnn hinders its performance, causing loss of white lines. U-Net’s results are intriguing, with many white regions indicating accurate delineation (Fig 10). Filtering improves U-Net’s performance, reducing spurious objects, contrary to its behaviour on other images. Trace Ridges maintains strictness, yielding cleaner delineations (Fig 14), albeit missing some key fibres. Graph based algorithm performs similarly with Trace Ridges, with filtering reducing spurious elements (Fig 14).

#### Disease mimicking ECM.

A visual examination of the disease mimicking ECM (DME) image (Fig 11) reveals notable insights. By looking at the data image, both Gaussian and DnCnn filtering visibly enhance image clarity. Edge Detection does not perform well. The main drawback of Edge Detection is that there are two lines for each fibre. The results show many blue lines, where many fibres were detected that are not there. Filtering notably enhances Edge Detection’s performance, particularly evident in the ROI (Fig 15), where DnCnn offers the most significant improvement. CT Fire performed better than Edge Detection, it also presented spurious objects. Gaussian and DnCnn filtering resulted in more non-present traces; this is easily viewed in the ROI (Fig 15). Scale Space displays fewer traces, benefiting from its multi resolution capabilities, but may merge separate fibres occasionally. Gaussian and DnCnn filtering have increased the number of fibres, but the latter has resulted in the long fibres separating. Twombli yields mediocre results, segmenting non-existent fibres, yet outperforming certain algorithms. Filtering enhances Twombli’s delineation, with Gaussian filtering notably improving the algorithms performance, this is evident in the number of white lines in (Fig 15). However, DnCnn leads to spurious lines. U-Net performs well, especially on the original image, but shows degraded performance on filtered images due to training on non-filtered data. The Graph based algorithm demonstrates robust performance, further improved with filtering, as seen in (Fig 15), where DnCnn enhances accuracy and cleanliness. Trace Ridges, akin to Scale Space, exhibits fewer traces, deliberately separating intersecting fibres for improved accuracy. Filtering enhances Trace Ridges’ performance, particularly evident in (Fig 15).

## Conclusions

In this paper we introduced *Trace Ridges*, a novel method for tracing fibre-like structures, compared against six existing methods (Edge Detection, CT Fire, Scale Space, Twombli, U-Net and Graph based) with three filtering options (no filtering, Gaussian filtering and DnCnn). Gaussian filtering is a traditional filtering technique that uses Gaussian kernels while DnCnn is a deep neural network. Images representing SHG of tumor bearing mouse mammary glands images, fluorescently labelled fibronectin images, Picrosirius red breast cancer collagen fibres images and disease mimicking ECM images. These images were used for evaluation, compromising both noisy and clean samples.

Manual ground truth delineations were created for each image and distance maps were used to quantify distance errors between the ground truth and algorithmic traces.

Trace Ridges exhibited the lowest total distance error, followed by Graph based and U-Net. Edge Detection achieved the lowest average distance error, attributed to its tendency to over-segment, thus its traces are always close to a fibre from the ground truth. Edge Detection was followed by Trace Ridges and Twombli. U-Net attained the lowest maximum distance error followed by Graph based and Edge Detection. Trace Ridges did not perform well in this metric as it favours brighter and longer fibres, so it is prone to have a higher singular maximum error.

Interestingly, filtering improved results for all algorithms except U-Net, which was trained on non-filtered patches. However, filtering enhanced U-Net’s performance specifically with the Breast Cancer Biopsy image.

Edge Detection was the fastest on average, followed by Trace Ridges and U-Net. Filtering generally expedited algorithms except for U-Net, although it improved processing time with the Breast Cancer Biopsy image.

Overall, Trace Ridges demonstrated superior performance, achieving the lowest total distance error, second-lowest average distance error, and second-fastest processing time. It offers rapid, automated tracing and extracts essential topological and morphological information, including fibre length, fibre count, orientation, circularity, and gap characteristics. Thus, Trace Ridges emerges as the preferred algorithm for tracing various fibre-like structures, including extracellular matrix and collagen fibres.
